# The Association of Poor Oral Health Parameters with Malnutrition in Older Adults: A Review Considering the Potential Implications for Cognitive Impairment

**DOI:** 10.3390/nu10111709

**Published:** 2018-11-08

**Authors:** Anastassia E. Kossioni

**Affiliations:** Division of Gerodontology, Department of Prosthodontics, Dental School, National and Kapodistrian University of Athens, Thivon 2, 11527 Athens, Greece; akossion@dent.uoa.gr; Tel.: +30-210-746-1246

**Keywords:** oral health, tooth loss, masticatory function, dental care, dietary habits, nutrition, dietary counselling, cognitive decline

## Abstract

Poor dental status and chewing deficiencies have been associated with cognitive decline. Altered dietary habits and malnutrition have been suggested as linking mechanisms. The aim of the present review was thus to investigate if poor oral health, and in particular tooth loss and impaired masticatory function, may affect dietary selection and nutritional intake in older adults, and moreover, to assess if prosthodontic dental care may improve nutritional status. Extensive tooth loss may impair masticatory function. Several studies in older populations have shown that severe tooth loss and masticatory impairment are associated with limited consumption of various food types (especially fruits and vegetables), increased consumption of sugary and easy-to-chew foods, and lower dietary intake of fibre and vitamins. However, these findings are not consistently reported, due to methodological variation among studies, potential adverse causalities, and the multifactorial nature of food choices. On the other hand, a few interventional studies revealed that prosthetic rehabilitation of missing teeth, when accompanied by dietary counselling, may improve dietary habits and nutritional intake. Further research is required to improve current knowledge of these associations. Under the limitations of the current study, a functional dental arch of natural or artificial teeth is important for maintaining adequate chewing efficiency and ability, but this only partly contributes to food choices and nutritional status. The multifactorial nature of food choices necessitates the interprofessional collaboration of dental professionals, dietetics practitioners, and primary care providers to improve dietary habits and nutritional intake.

## 1. Introduction

Oral health is important for well-being as it is associated with pain, infection, xerostomia, problems with chewing, swallowing, speaking, smiling, communicating, and socializing [1,2]. Oral disease is common in older adults, and involves tooth loss, poor oral hygiene (increased amounts of soft and mineralized deposits found on teeth and denture surfaces), high prevalence of dental caries and periodontal disease, defective prosthetic appliances or absence of prosthetic rehabilitation, hyposalivation, and various oral lesions, often associated with denture-wearing but also with precancerous or cancerous states [1,2,3,4]. Most oral conditions can be prevented, or efficiently managed, if detected early [5].

The oral cavity is the first part of the digestive tract, responsible for biting the food, chewing, adding saliva for bolus formation, and transporting it into the stomach [6,7]. Deficits in any of these stages may impair eating. Some poor oral health indicators, and particularly tooth loss and chewing deficiency, have been associated with nutritional impairment [8,9,10,11,12].

The potential association of some poor oral health parameters with cognitive decline has recently gained increasing attention [13,14,15,16,17,18]. Two of the most commonly reported ones are tooth loss [13,14,16] and masticatory impairment [15,18]. A systematic review investigating the association between mastication and cognitive status, recorded by various cognitive function tests (Mini-Mental State Examination (MMSE), Montreal Cognitive Assessment, digit-symbol substitution, etc.), revealed that poorer mastication was associated with lower cognitive function in 15 of the 17 cross-sectional studies, and a steeper decline in five of the six prospective studies [18]. The same systematic review recorded that poorer mastication was a significant risk factor for having dementia or mild memory impairment in four of five cross-sectional studies and for the incidence of dementia or mild memory impairment (MMI) in four of five prospective studies [18]. Potential linking mechanisms are altered dietary habits due to impaired oral health, and malnutrition [17,18,19].

Decreased consumption of fibre and micronutrients and increased consumption of softer, easy-to-chew foods rich in saturated fats and cholesterol may be associated with cognitive impairment either through micronutrient deficiencies (i.e., vitamin B12, thiamine) or by adopting unhealthy diets, increasing the risk of stroke and dementia [17]. It should be noted that adherence to the Mediterranean diet has been associated with reduced risk for mild cognitive impairment, dementia, and particularly Alzheimer’s disease [20], but other diets, adapted to regional cultures, are also being investigated, showing promising results [21]. Moreover, mastication might be a protective factor for cognitive decline, as it is related to increased blood flow in specific brain areas (the cerebral cortex, cerebellum, thalamus, and hippocampus) [19,22]. However, the nature and causality of these associations is yet to be determined as there are many confounding factors [14,16,18].

Based on the hypothesis that tooth loss and chewing problems may be associated with cognitive decline, mainly through altered dietary habits and malnutrition, the present review aims to investigate if oral parameters, and in particular tooth loss and impaired masticatory function, may affect dietary selection and nutritional intake in older adults. The aim was moreover to assess if prosthodontic dental care may improve nutritional status.

The null hypotheses are that: (1) poor oral health, and particularly poor dental status, negatively affect masticatory function; (2) poor oral health, and particularly poor dental status and masticatory impairment, induce altered food selection and malnutrition; and (3) prosthodontic care improves nutritional status.

## 2. Does Poor Oral Health Affect Masticatory Function?

Masticatory function can be evaluated objectively using various tests (chewing efficiency tests, biting force, etc.), and subjectively using questionnaires. While objective chewing problems are directly related to fewer number of teeth [7,23], subjective chewing difficulties, particularly when eating hard food, start when there are fewer than 20 teeth in the mouth [8,24]. The subjective perception of masticatory ability is generally more optimistic than the objective one [24], but may be more crucial for the individual’s dietary choices.

Apart from the actual number of remaining teeth, their arrangement in the dental arch is also important for chewing performance; therefore, a more sensitive indicator for chewing problems is the number of occluding tooth contacts or functional tooth units or occluding tooth units, particularly in the area of premolars and molars. A systematic review investigating the association between functional teeth units and chewing ability in older adults, based on self-reporting, has shown that chewing problems increased with decreasing numbers of posterior functional tooth units, when the remaining teeth were unevenly distributed, or when there were tooth-bounded spaces [24].

More than three posterior functional tooth units are necessary to keep the functional ability of the stomatognathic system in older age [25,26,27]. Individuals with fewer than three posterior functional tooth units have a reduced masticatory performance (by 30–40%), while partial dentures only partially compensate for the reduced masticatory function [28]. On the other hand, individuals with more than 20 teeth and/or more than five posterior functional tooth units may chew a large variety of foods [8].

Tooth loss is still a common finding in older age, with wide variation between countries. The global prevalence of edentulousness in people aged 65–74 years in upper/middle-income countries is still high (35%) [2], while the mean number of remaining natural teeth is lower than 20 in most countries, particularly in those over 75 years of age [29]. The rate of edentulousness in people over 65 years in Europe ranges from 7% to 45% [30]. These epidemiological findings reveal that at least in higher income countries few older people have a natural functional dentition of 20 teeth, with potential negative implications for their chewing function if dental care has not be provided

Apart from tooth loss, other parameters of the stomatognathic system may affect masticatory ability, such as hyposalivation and swallowing disturbances.

Hyposalivation, which is very common in old age, adversely affects masticatory function. Saliva secretion is closely related to enabling bolus formation, sliding of food through the oesophagus, and initiation of digestion procedures [6,7,31]. Saliva secretion increases when eating, and its absence may disturb bolus preparation and swallowing [6]. Most authors agree that ageing per se does not induce salivary dysfunction that is mainly related to general medical conditions (i.e., Sjögren syndrome, depression, Parkinson’s disease, dehydration, anaemia, diabetes mellitus, etc.) and xerostomic medications (i.e., antidepressants, antihypertensives, antihistamines, etc.) [32]. Another important function of saliva is taste sensitivity [31], which is often impaired in older age, leading to undernutrition and weight loss.

Swallowing problems may also impair the ability to eat the amount and quality of foods that meet nutritional needs [33,34]. Apart from various general medical conditions, local factors such as tooth loss and absence of dentures inhibiting masticatory ability, may disturb the execution of smooth swallowing [33,35]. On the other hand, denture-wearing in edentulous patients may improve swallowing function [33]. Alterations in the motor function of the tongue could further induce eating and swallowing problems causing undernutrition [7,34].

Based on the existing evidence, many oral health parameters, including extensive tooth loss without prosthetic rehabilitation, hyposalivation, swallowing disorders, and impaired tongue movements, may impair masticatory function. Therefore, the first null hypothesis is accepted.

## 3. Does Poor Oral Health Affect Food Selection and Nutritional Intake?

The effect of oral factors, particularly tooth loss, on dietary preferences and nutritional intake has been investigated in numerous cross-sectional and prospective studies using various measures (self-administered questionnaires, oral health-related quality of life tools, Mini Nutritional Assessment (MNA) questionnaires, haematological data, etc.).

### 3.1. Dental Status and Food Selection

Using patient interviews, several studies have associated tooth loss and denture-wearing with limited consumption of specific food types such as meat, fruits and vegetables and increased consumption of sugary products and soft easy-to-chew foods [8,24,36,37,38,39,40]. On the other hand, other studies showed limited dietary restrictions related to tooth loss and chewing difficulties [41,42].

Individuals with self-perceived ill-fitting dentures had lower dietary quality scores consumed fewer fruits and vegetables, and had a lower variety of foods in their diet as compared with participants with 18 or more teeth [38]. However, those with self-perceived good fitting dentures had diets that were not different from subjects with 18 or more teeth [38], indicating that successful dental treatment may improve dietary selection. Electromyographic studies have shown that experienced denture wearers make necessary functional adjustments to the masseter muscle neuromuscular activity in order to chew hard food, while the role of the powerful periodontal mechanoreceptors, lost after tooth extractions, may be taken over by other oral receptors [43].

On the other hand, food choices greatly depend on the local culture. Greeks with fewer teeth did not exclude from their diet any food types that were difficult to eat [41,42] as compared to English study participants [37]. Participants with fewer teeth or edentulous denture wearers used various methods of food preparation which helped them to eat most food types. Such examples are mincing the meat, cooking vegetables and greens in olive oil, boiling chicken, selecting easy-to-chew fruits such as oranges, melons, and grapes, and cutting apples into small pieces, etc. [41,42]. Moreover, local restaurants and family cookers prepare foods that are easy to chew for older edentate people [44]. Actually, older Greeks had dietary habits closer to the traditional Mediterranean diet than the younger study participants, irrespective of their dental status, indicating the multifactorial nature of food choices [41]. Likewise, middle-aged patients treated with conventional complete dentures in Canada, while reporting some chewing difficulties for hard foods, did not exclude them from their diet [45]. They often cut them down into small pieces or used pureed meats or raw fruits and vegetables instead [45].

### 3.2. Dental Status and Nutritional Intake

A number of cross-sectional surveys in large samples of older populations revealed that edentate people and those with few teeth had lower nutritional intake [8,9,10,11,12], mainly in vitamins and dietary fibre [39]. Individuals with more than 20 natural teeth consumed more of the majority of nutrients [8], while denture wearers had significantly lower serum levels of vitamins C and E, beta carotene, folate, lutein, and lycopene/zeaxanthin compared with the dentate group with more than 18 teeth [9]. Moreover, 22% of the variability in the Mini Nutritional Assessment (MNA) could be explained by dental status [11].

A systematic review and meta-analysis investigating the association between oral health in individuals older than 60 years and nutritional status has shown that malnourished people presented 0.14 less teeth on average when compared to well-nourished individuals [46]. However, the risk of edentulism or using a prosthesis did not reveal any differences between malnourished and well-nourished persons [46]. Other studies revealed limited or no associations between dental status and malnutrition [47,48].

Tada and Miura [39] in their systematic review on the association of mastication with food and nutrient intake in independent older adults noticed that the studies that did not reveal a positive association between mastication and food and/or nutrient intake were carried out in developed countries, where more processed food is consumed. The authors attributed this finding to cultural differences between countries and the consumption of processed food [39].

Inconclusive evidence is also recorded in studies investigating the association between dental status and body mass. Chewing difficulties may lead to lower food intake causing undernutrition and weight loss or to chewing easy-to-chew food, rich in fat and sugars, leading to weight gain. Data from a prospective study using data from the Survey on Health, Well-being and Aging in Brazil (SABE), has shown that the risk of weight and waist circumference loss was higher among edentulous community dwelling older adults than among dentate ones [49]. The study of Lee et al. [50] in older Koreans showed that Body Mass Index (BMI) was positively associated with the number of missing teeth in females. The National Diet and Nutrition Survey (NDNS) in independent adults aged 65 and over in the United Kingdom revealed that having functioning natural dentition of more than 20 teeth increased the likelihood of having a normal BMI, while having few natural teeth or being edentulous was associated with a greater risk of being underweight or being obese [51]. Cultural or methodological variation may explain these discrepancies.

A systematic review aiming to explore whether tooth loss affects dietary intake and nutritional status, based on eight longitudinal studies, revealed contradicting results, but the quality of studies was considered as fair (1) or poor (7) and more high-quality longitudinal studies should be designed [52].

Apart from tooth loss, other oral health parameters have also been associated with malnourishment, such as xerostomia, and swallowing and taste difficulties [12,34,53]. Individuals complaining of xerostomia were 3.49 times more likely to be malnourished than others, based on MNA scores [12]. Hospitalized patients with swallowing difficulties were almost five times more malnourished, and those with taste difficulties had 2.5 times more risk to be malnourished [53].

### 3.3. Oral Health and Nutritional Intake in Institutionalized Older Persons

The association of oral health with nutritional status in institutionalized older persons has been investigated in a number of studies and was further explored through systematic reviews. Edentulousness, chewing problems, hyposalivation, dysphagia and problems with the tongue have been associated with weight loss, low BMI, and malnutrition [8,54,55]. Nutrient intake in both dentate and edentulous older institutionalized persons in the United Kingdom was poor and similar to that of edentulous people living in the community [8].

Most studies agree that although there is some evidence of an association between oral health and malnutrition in nursing home residents, this strength is weaker than in community-dwelling adults [8,46,54,55], but causality is very difficult to identify due to various confounders [54]. Moreover, various methodological issues were raised, such as cross-sectional design, multiple definitions of malnutrition, and lack of multivariate analyses [54,55].

The reason for the limited variation in nutritional intake in relation to dental status in the institutions has been attributed to the adaptation of the diet to those with poorer masticatory ability [8], and to the poor general condition of institutionalized individuals (i.e., dementia, depression, polypharmacy, immobility, advanced age) that directly affect nutritional status [46]. Indeed, a cross-sectional study in older patients with dementia did not reveal any significant associations of dental state and chewing efficiency with nutritional state, but chewing efficiency was more strongly associated with cognitive impairment than with tooth loss [56]. Cognitive decline may affect nutritional intake and oral motor coordination, and impair the ability to maintain efficient oral hygiene and access dental care [14,16]. Patients with dementia have poorer oral hygiene compared to healthy individuals, fewer teeth, more dental caries, and increased prevalence of hyposalivation and taste disturbances due to medications; they often suffer from oral dyskinesias and impaired chewing ability, affecting eating and, in the later stages of the disease, they usually cannot use their dentures [19,33,57].

Based on the current literature, most studies recorded an association of oral factors such as tooth loss and denture-wearing with altered dietary choices and poor nutritional status, but these findings are not consistent. Methodological variation and the multifactorial nature of food choices may be responsible for these inconsistencies. Even when a strong association is recorded, the causality may be unclear due to numerous confounding factors and adverse effects, present in older people, such as physical and mental conditions, ability for self-care, education, socioeconomic status, impaired taste and odour, hyposalivation, impaired oral motor function, functional ability to buy and prepare food, personal preferences, local culture, loneliness, marital status, institutionalization, ethnicity, behavioural variables, religion, etc. [10,11,12,41,53,54,56]. On the other hand, malnutrition may adversely affect the health of oral tissues. Such examples are deficiencies in vitamins A, C, E, copper, iron, zinc and non-nutrient antioxidants that may depress anti-inflammatory and immune response of oral soft tissues, or limited protein intake that may compromise response to infection and wound healing [58].

The above findings reveal that although poor dental status is associated with chewing difficulties, its effect on food selection and nutritional intake is still open to debate. Therefore, under the limitations of the present study, the second null hypothesis is rejected and more studies are necessary to clarify this issue.

## 4. Does Prosthodontic Care Improve Nutritional Intake?

Most intervention studies did not record any significant improvement in nutritional status after prosthetic rehabilitation of missing teeth [39,59,60]. One study recorded significant improvement only using the MNA score but not its short form (MNA-SF) [61]. 

A number of studies have further investigated whether the type of prosthetic treatment affected nutritional intake. They compared any variation in nutritional status between patients rehabilitated with dental implants and those rehabilitated with conventional dentures, but again no significant differences were recorded [45,60,62,63]. Although denture wearers had diminished masticatory ability compared to implant patients, they did not avoid any specific food types [45].

However, when dietary consultation was offered together with prosthetic rehabilitation, dietary habits significantly improved, irrespective of the type of prosthesis [64,65,66]. Bradbury et al. [64] combined prosthetic treatment with dietary counselling by a nutritionist, delivering a tailored written package to 58 edentulous patients in the United Kingdom, and 6 weeks after denture placement recorded greater increase in fruit/vegetable consumption compared to a control group that was not offered any dietary counselling. Likewise, in another UK study, nutritional advice improved nutritional intake in both samples using with either conventional complete dentures or implant-supported mandibular overdentures, with modestly better improvement in implant patients [65]. An interesting finding was that dietary improvements decreased over time, indicating that ongoing dietary intervention is needed [65].

Under the limitations of the existing evidence, prosthodontic rehabilitation alone does not necessarily improve nutritional intake, and the third null hypothesis should be rejected. On the other hand, it appears that dietary counselling for both patients and their caregivers along with prosthodontic treatment may improve nutritional status.

## 5. The Need for Interprofessional Collaborative Practice to Improve Diet and Nutrition in Older People

Conservative and prosthodontic dental care focusing on preservation and rehabilitation of strategic parts of the dental arch may improve chewing efficiency and ability, helping people to chew a large variety of foods. However, this may not be enough for improving dietary preferences and nutritional status, due to the multifactorial nature of food choices. Dentists should include nutritional screening and counselling during dental assessment, dental care provision, and recalls, especially in patients -and their caregivers- with severe tooth loss, and provision of conventional or implant-supported dentures. A further collaboration with a dietetics practitioner may be necessary to improve diet in patients with high risk of malnutrition, particularly the frail and care dependent ones, living in the community or in institutions.

It should be noted that a standard dental procedure after insertion of new complete dentures is offering practical advice to patients on eating modifications to prevent their dislodgment during function and improve functional adaptation and patient satisfaction [67].This advice includes: starting with small amounts of soft food placed on both sides of the mouth and later adding harder food in the meals; avoiding biting with the front teeth as this may dislodge the dentures; chewing with the back teeth on both sides simultaneously; trying to eat all types of foods with the necessary preparation (for example by cutting the meat, hard fruits, raw vegetables and salads into small pieces before eating, well-cooking or mincing the meat, mashing vegetables and fruits, boiling or cooking raw vegetables, moistening the bread and rusks in water before eating), trying to avoid sticky foods (white rice, white bread, sticky sweets) that may dislodge the dentures, and trying to avoid foods containing small seeds (e.g., sesame bagels, kiwi, tomatoes) or removing the seeds before eating, as they may get under the dentures and cause irritation. Moreover, it is stressed that it may take some time for the stomatognathic system to adapt to using the new appliances and be able to eat most types of food without discomfort.

On the other hand, non-dental health care providers should incorporate oral screening within patient assessment to record any oral disease or defects in the existing prostheses that may cause eating problems [4]. The Academy of Nutrition and Dietetics recommended oral screening within nutrition care process by dietetics practitioners, and a close collaboration with the dentist when oral health problems are identified [58].

## 6. Implications for Cognitive Decline Prevention

Considering the potential beneficial influence of a healthy diet on cognitive performance, any factors affecting dietary choices should be addressed. Good oral health, and particularly a functional dental arch of natural or artificial teeth is important for maintaining adequate chewing efficiency and ability, and being able to eat a large variety of healthy foods. However, it should be taken into consideration that there is a large number of personal, socio-economic, medical, cultural and other modifiers in food choices.

Therefore, prevention measures for good oral health throughout the lifespan, dental care provision, when necessary, offered with tailored nutritional assessment and counselling, and systematic oral screening during medical and dietary assessment may offer multiple health benefits.

## 7. Conclusions

As a healthy diet may have a beneficial effect on cognitive performance, the role of oral health, and particularly dental status, on dietary choices is very important. Severe tooth loss and masticatory problems partly contribute to restricted dietary choices and poor nutritional status of older adults, due to multiple confounders. There is also increasing evidence that prosthodontic treatment when offered with tailored nutritional advice may improve the nutritional status of patients.

The interprofessional collaboration of dental professionals, dietetics practitioners, and primary care providers is required, incorporating oral and dietary screening in daily practice, providing dietary advice, preventing and treating oral disease, and rehabilitating strategic parts of the dental arch to improve chewing ability and dietary habits.
